# The Structure of Phosphorylase Kinase Holoenzyme at 9.9 Å Resolution and Location of the Catalytic Subunit and the Substrate Glycogen Phosphorylase

**DOI:** 10.1016/j.str.2008.10.013

**Published:** 2009-01-14

**Authors:** Catherine Vénien-Bryan, Slavica Jonic, Vasiliki Skamnaki, Nick Brown, Nicolas Bischler, Nikos G. Oikonomakos, Nicolas Boisset, Louise N. Johnson

**Affiliations:** 1 Laboratory of Molecular Biophysics, Department of Biochemistry, University of Oxford, South Parks Road, OX1 3QU Oxford, UK; 2 IMPMC-UMR 7590, CNRS, Universités Paris 6 et Paris 7, IPGP, 75252 Paris, France; 3 Institute of Organic and Pharmaceutical Chemistry, The National Hellenic Research Foundation, 48 Vassileos Constantinou Avenue, 116 35 Athens, Greece

**Keywords:** PROTEINS, SIGNALING

## Abstract

Phosphorylase kinase (PhK) coordinates hormonal and neuronal signals to initiate the breakdown of glycogen. The enzyme catalyzes the phosphorylation of inactive glycogen phosphorylase b (GPb), resulting in the formation of active glycogen phosphorylase a. We present a 9.9 Å resolution structure of PhK heterotetramer (αβγδ)_4_ determined by cryo-electron microscopy single-particle reconstruction. The enzyme has a butterfly-like shape comprising two lobes with 222 symmetry. This three-dimensional structure has allowed us to dock the catalytic γ subunit to the PhK holoenzyme at a location that is toward the ends of the lobes. We have also determined the structure of PhK decorated with GPb at 18 Å resolution, which shows the location of the substrate near the kinase subunit. The PhK preparation contained a number of smaller particles whose structure at 9.8 Å resolution was consistent with a proteolysed activated form of PhK that had lost the α subunits and possibly the γ subunits.

## Introduction

Protein phosphorylation and dephosphorylation reactions are major control mechanisms in eukaryotic cells. Skeletal muscle phosphorylase kinase (PhK) was the first protein kinase to be recognized by function and to be purified over five decades ago (Fischer and Krebs, 1955; Krebs et al., 1964). PhK is a key enzyme in the control of glycogenolysis. Intracellular glycogen stores are used primarily to maintain blood-glucose homeostasis during fasting and as a source of energy for muscle contraction. PhK integrates signals from different signal pathways—hormonal messengers (adrenaline), neuronal stimuli (Ca^2+^), and metabolic signals (e.g., adenosine diphosphatase [ADP] levels)—to produce rapid mobilization of glycogen stores. PhK phosphorylates inactive glycogen phosphorylase b (GPb) on a single serine, Ser14, converting it to active phosphorylase a (GPa). Active phosphorylase catalyzes the phosphorolysis of glycogen to release glucose-1-phosphate. All eukaryotic protein kinases share a common catalytic core, which catalyzes the transfer of the γ-phosphate from a nucleoside triphosphate donor to the side-chain hydroxyl of a serine, threonine, or tyrosine in substrate proteins, but each kinase recognizes only specific substrates and each is regulated by a distinct mechanism.

PhK is one of the largest and most complex of the protein kinases (Brushia and Walsh, 1999; Heilmeyer, 1991). It is composed of four types of subunit, with stoichiometry (αβγδ)_4_ and a total molecular weight (MW) of 1.3 × 10^6^. The α (138 kDa, 1237 amino acids) and β (125 kDa, 1092 amino acids) homologous subunits are regulatory inhibitory subunits. Autophosphorylation or phosphorylation of these subunits by cyclic adenosine-monophosphatase-dependent kinase in response to hormonal stimulation leads to activation of the kinase. ADP binds to the β subunit with high affinity and also stimulates activity. Neuronal stimulation is effected by Ca^2+^ ions, which bind to the intrinsic calmodulin (δ) subunit (16.7 kDa, 148 amino acids), thus coupling muscle contraction with energy production. PhK differs from most other calmodulin-regulated enzymes in that the δ-calmodulin subunit is tightly associated in the holoenzyme in the absence of calcium. The γ subunit (44.7 kDa) is the catalytic subunit. It is composed of a kinase domain (residues 20–276) and a regulatory calmodulin-binding autoinhibitory domain (residues 298–396). PhK is one of several kinases that do not require phosphorylation on the kinase activation segment for activity. When isolated, the kinase domain is constitutively active. It has a glutamate in place of the more typical phosphorylatable serine, threonine, or tyrosine in the activation segment (Lowe et al., 1997; Owen et al., 1995). Hence, the role of the regulatory α, β, and δ subunits is to restrain, either directly or indirectly, the activity of the kinase domain until signals are received that relieve the inhibition.

Structural data are available for the kinase domain and for calmodulin, but little is known of the homologous α and β subunits. Recent sequence comparisons have suggested that the N-terminal regions residues 1–436 (α subunit) or residues 40–477 (β subunit) share sequence similarities with family 15 glycosyl hydrolases (Pallen, 2003), whereas the C-terminal region residues 1066–1237 (α subunit) and 918–1093 (β subunit) share similarities with the calcineurin B-like proteins (Carriere et al., 2008) that are composed of two pairs of EF hands and that might contribute to the regulation by Ca^2+^. In addition, a leucine zipper motif has been identified in the α subunit, but not the β subunit, for residues 833–854 (Ayers et al., 1999).

The activation of PhK is complex, with several different layers of control. In vitro pH is a further regulator. Inactive PhK assayed in the presence of calcium exhibits low activity at pH 6.8. Activity is markedly increased at pH 8.2, but still requires the presence of calcium. Activation of PhK by phosphorylation or limited proteolysis results in an increase in activity at pH 6.8 but little change in activity at pH 8.2 (Newsholme and Walsh, 1992).

In order to understand the structural basis of the mechanisms involved in control of PhK through protein-protein interactions and by phosphorylation, a structure of the whole enzyme is required. Intact PhK has been difficult to crystallize for X-ray studies, but it has proved amenable to single-particle analysis by electron microscopy. We previously determined the three-dimensional (3D) structure of PhK at 22 Å resolution using negative stain electron microscopy and the random conical tilt method (Venien-Bryan et al., 2002). The 222 symmetric structure shows a butterfly-like arrangement with two wing-like lobes connected by two oblique bridges. We also determined the structure of PhK decorated with GPb at 28 Å resolution in the presence of activating Ca^2+^, Mg^2+^ at pH 8.2 showing that GPb bound toward the end of the lobes. At the same time, Nadeau et al. (2002) compared the negatively stained structures of inactive PhK at pH 6.8 in the absence and presence of calcium, and showed that calcium promotes a redistribution of density throughout the lobe and bridge regions without perturbing the overall dimensions. This model has been further improved and elaborated in subsequent cryo-electron microscopy (cryo-EM) studies at 25 Å resolution and small angle X-ray scattering to detect calcium induced structural changes (Nadeau et al., 2005; Priddy et al., 2005).

Here, we present the structure of calcium activated PhK at pH 8.2 at subnanometer resolution using frozen unstained specimens and cryo-EM. In the 3D model we located the γ catalytic subunit (PhKγ_t_ residues 1–298), whose kinase domain structure has been solved by X-ray crystallography (Lowe et al., 1997; Owen et al., 1995). The high-resolution cryo-3D single-particle studies have been extended to produce a 3D model of the PhK/GPb complex. Visualization of this complex allows us to elaborate in greater detail on the interaction and contact between PhK and its substrate. Structures of glycogen phosphorylase in both its active and inactive state are known from X-ray studies (Barford and Johnson, 1989). We also present the structure of the activated proteolysed PhK, which might have a physiological role in apoptosis (Hilder et al., 2005).

## Results

### Purification and Activity Measurements

**Figure 1:**
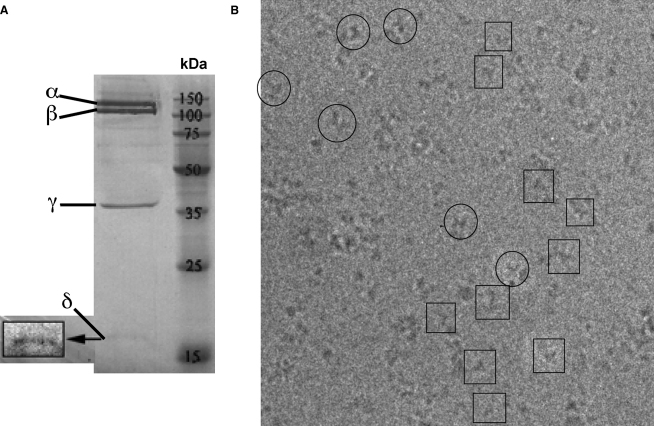
Characterization of PhK (A) PhK electrophoresis 10% SDS-PAGE gel after the last purification step. (B) Cryo-electron micrograph of PhK. The characteristic butterfly view is highlighted with circles. Other views are highlighted with squares.

SDS-PAGE electrophoresis of PhK after the last purification step showed four bands corresponding in molecular size to the four subunits of PhK and indicated pure enzyme had been isolated (Figure 1A).

The PhK was shown to be functionally active at pH 8.2 in the presence of calcium against GPb substrate with k_cat_ values of 134 s^−1^ and 97 s^−1^ and K_m_ 9.3 μM and 42 μM versus GPb and ATP substrates, respectively, as the variable substrate (Table 1A). PhK holoenzyme had approximately 2.2-fold higher k_cat_ than the kinase domain PhKγ_t_ with both GPb and ATP as the variable substrate. Values of K_m_ for GPb were similar (9.2 μM and 8.5 μM, respectively). K_m_ values for ATP showed a 2-fold difference between the PhK holoenzyme and PhKγ_t_ (41.5 μM and 82.1 μM, respectively). The kinetic parameters suggest that the activity of the kinase, when incorporated into the holoenzyme, has slightly different properties that make it a more efficient enzyme.

Comparison between specific activities for the calcium-activated states of PhK at pH 8.2 (5.2 μmol/min/nmol) and at pH 6.8 (0.65 μmol/min/nmol) (Table 1B) shows that the ratio for 6.8/8.2 is 0.125. This ratio is higher than the value of ∼0.05, which is usually accepted as indication for unmodified PhK (Newsholme et al., 1992). It has been shown that activation by proteolysis results in a pH 6.8/8.2 activity ratio between 0.2 and 0.9 (Newsholme et al., 1992). This is discussed further below.

### 3D Reconstruction Volume of PhK by Cryo-EM at 9.9 Å Resolution

**Figure 2:**
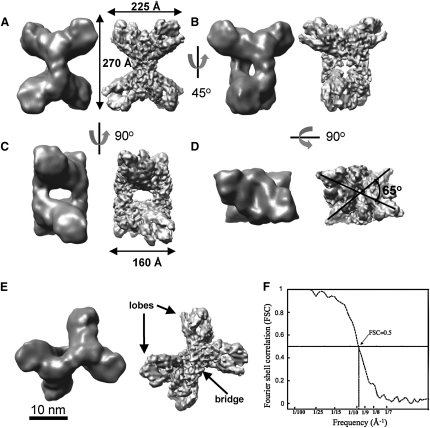
The 3D Structure of Phosphorylase Kinase Surface representations of PhK calculated from 18,123 selected frozen particles in different orientations. Cryo-EM and negatively stained (see Venien-Bryan et al., 2002) 3D volumes are shown (right and left respectively). (A) Front butterfly orientation. (B) Chalice orientation, from a rotation of 45° of the butterfly orientation about the vertical axis. (C) Tetrad orientation, from a rotation of 90° of the butterfly orientation about the vertical axis. (D) A 90° rotation of the butterfly orientation about the horizontal axis showing the rotation angle between the two lobes. (E) Oblique view. (F) Determination of the resolution of the final cryo-3D reconstruction of PhK. The resolution is 9.9 Å as estimated by 0.5 FSC cut-off. The bar scale is 10 nm.

PhK was prepared under conditions (pH 8.2 and in the presence of calcium) where it is known to be in the active conformation as indicated from the kinetic studies. PhK was imaged in cryoconditions. A range of recognizable particle shapes could be distinguished in the electron micrographs as illustrated in Figure 1B. The 3D structure was calculated with 18,123 defocus-corrected particles using SPIDER (Frank et al., 1996) (Figures 2A–2E) and refined to 9.9 Å resolution as assessed from the 0.5 Fourier-shell correlation (FSC) cut-off (Figure 2F). The contour level for surface display was chosen so that it encloses a volume corresponding to the known molecular mass (1.3 × 10^6^ Da) as calculated with a protein partial specific volume of 0.84 Da/Å^3^. A gallery of the class averages of PhK and the corresponding reprojections of the 3D map is shown in Figure S1 (available online).

The comparison with our previous model obtained from negatively stained PhK at 22 Å resolution is shown in Figure 2. The overall shape of the 3D models (negatively stained and cryo) is the same, but the cryo-3D model shows a better-resolved structure, which has lost its smooth appearance and exhibits finer details. The dimensions of the overall structure are 270 × 225 × 160 Å. The butterfly-like view (Figure 2A) shows that PhK is made of two opposing lobes that are held together by two short oblique cross bridges. The dimensions of the lobes in the projection are 225 Å × 110 Å, and the bridges are 55 Å in length and 45 Å in width. Figures 2B and 2C show the chalice and the tetrad orientations. The hole inside the tetrad structure has a cross section of 35 × 85 Å. The relative orientation of the lobes can be seen after a 90° rotation about the horizontal axis of the butterfly orientation (Figure 2D). The angle between the lobes is 65°, as observed previously (Venien-Bryan et al., 2002).

The high-resolution (9.9 Å; 18,123 particles) cryomodel of active PhK is consistent with the broad features noted in the lower-resolution (25 Å; 4,878 particles) cryomodel of inactive PhK (Nadeau et al., 2005), but there are two main differences. These concern the angle between the lobes and the nature of the bridges. These features have been described as the most variable in previous reconstructions (Nadeau et al., 2005). The angle between the lobes observed here (65°) differs from the value (90°) noted by Nadeau et al. (2005). In previous negatively stained images, an angle of 68° had been noted which the authors conclude was a result of distortions induced by the stain. However in our present cryoimaging the enzyme has been preserved in the vitreous ice and there should be no distortions. In the present model we observe just two connecting bridges (Figure 2B), whereas Nadeau and colleagues reported that the two bridges resolved to four bridges with asymmetric connections to the two lobes. These differences between the two cryoimages give intriguing clues to the possible conformational changes that occur on activation of PhK that will be the subject of a future collaborative comparison.

### Location of the γ Subunit

**Figure 3:**
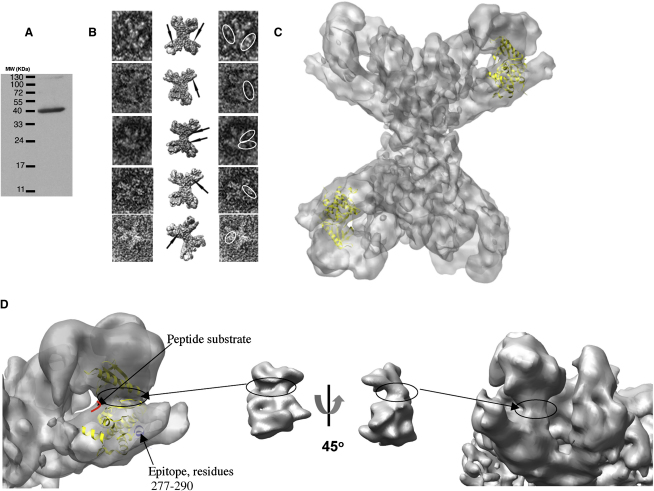
Location of γ-Catalytic Subunit into the 3D Model of PhK (A) Visualization of the γ subunit of PhK by immunoblot analysis. (B) Immunolocalization of the γ subunit into the 3D EM model. Five selected particle images negatively stained with the γ subunit antibody (left) are depicted together with the corresponding view of PhK structure (middle) where the position of the antibody label is indicated by an arrow; and the same particle images with the antibodies highlighted (right). (C) Fitting of the cryo-EM volume with two copies of the truncated form of the γ catalytic subunit of PhK (PHKγ_t_ PDB 2PHK) in yellow. The other two copies of PhKγ_t_ are behind and obscured. (D) Left close-up view of the PhKγ_t_ fitted onto the 3D PhK model. Residues 277–290 used as epitopes for the antibody anti-γ are shown in blue. The substrate peptide in complex with PhKγ_t_ in the active site is shown in red. Middle atomic model of PhK_γt_ converted to SPIDER format and filtered to a resolution comparable to the PhK 3D cryo-EM volume PhK (front and side views). Right close-up side view of the PhK. The cleft is highlighted with circles.

We have raised polyclonal antibodies against the γ kinase subunit peptide with the sequence (TAEEALAHPFFQQY residues 277–290). Immunoblot assays of the binding of the anti-γ antibody to denatured PhK show that these antibodies recognize the γ subunit (Figure 3A). Wilkinson et al. (1997) used enzyme-linked immunosorbent assay (ELISA) to show that a similar antibody raised to the same epitope recognizes the intact holoenzyme. Figure 3B shows a gallery of negatively stained PhK labeled with antibody against the γ subunit. The antibodies appear to bind at the internal side of the lobes. This result is consistent with the results of (Wilkinson et al., 1997) who observed a similar location with a monoclonal antibody directed against the same region of the γ subunit. Examination of about 600 images showed that about 20% of the PhK particles had no antibody bound, about 60% had one antibody bound, 16% had two antibodies, and 3% had three antibodies. The reasons for these stochastic observations are not clear. It is possible that the dual labeling with antibodies prevents simultaneous binding of the antibody complexes on adjacent lobes of the PhK molecule or that the epitope (residues 277–290) is not completely accessible for antibody binding. Occasionally PhK dimers or higher aggregates associated with the antibody were observed, but these were not numerous.

To determine more precisely the position of the γ catalytic subunit, we fitted the crystal structure of PhKγ_t_ (residues 1–298) (Lowe et al., 1997; Owen et al., 1995) into the 9.9 Å cryo-EM model using a rigid-body approach. Three different programs were used (see Supplemental Data), and each gave the same location of the PhKγ_t_ into the 3D model (Figure 3C). In this orientation of PhKγ_t_ into the 3D EM model, we note that the epitope against which the antibodies were raised (Figure 3D, in blue) is consistent with the position of the binding of the antibodies as seen in the negatively stained complexes.

The PhK γ subunit catalytic domain has the classical bilobal protein kinase structure. A smaller N-terminal lobe (residues 14–107), formed principally from β sheet, is connected to a larger C-terminal lobe (residues 110–292), formed mainly from α helices. The two lobes are joined by a hinge region around residues Lys108 and Gly109. The atomic models of PhKγ_t_ were converted to SPIDER format and filtered to a resolution comparable to the PhK 3D cryo-EM volume. The characteristic bilobal shape of PhKγ_t_ is shown in two orientations: front and side views (Figure 3D, middle). The N-terminal lobe of the PhKγ_t_ is the upper part of the molecule and the C-terminal lobe is the lower part. The cleft between these two domains is highlighted with circle. The cleft is also highlighted with circle on the 3D model of PhK (Figure 3D, left and far right). It appears that the bilobal structure with a definite cleft was a defining feature that guided all three programs for the modeling to ft PhKγ_t_ into this characteristic feature of the 3D EM model. The programs were used without any manual prefitting of the PhKγ_t_.

The PhKγ_t_ X-ray structure had been determined with a substrate analog peptide bound. The substrate peptide is shown in red in the magnified view of the site (Figure 3D, left). This defines the catalytic site of the kinase and the site at which the substrate, glycogen phosphorylase, should be recognized. In the crystal structure of PhKγ_t_ in complex with a substrate peptide, the kinase domain formed dimers across a two-fold crystallographic axis in a head-to-tail arrangement with part of the peptide substrate also involved in contacts across the two-fold axis (Lowe et al., 1997). However, in the fit of the domain to the PhK holoenzyme, the location toward the ends of the lobes indicates that the kinase domain is not involved in a homo-dimer interaction. This also implies that the association of PhK holoenzyme with GPb is unlikely to exploit the two-fold symmetry of the GPb dimer.

### 3D Reconstruction Volume of PhK/GPb by Cryo-EM

The PhK/GPb complex was formed in solution before deposition on the electron microscope grid. Divalent cations Mg^2+^ and Ca^2+^ were included in the buffer, because these enhance the affinity of PhK for GPb by about 8-fold (Xu et al., 1996). Quantitative assessment of the affinity of the multivalent proteins PhK tetramer (MW 1.3 × 10^6^) and GPb dimer (MW 1.94 × 10^5^) is not easy. The PhK/GPb complex was analyzed by gel filtration on a Superose 6 column, but the results were inconclusive. The K_m_ for the PhK phosphorylation of GPb is 9 μM (Table 1). For the truncated γ kinase subunit of PhK and for some other protein kinases, the kinetic constant is close to the dissociation constant (Skamnaki et al., 1999). Hence, K_d_ for PhK/GPb could be ∼9 μM. However, Xu et al. (1996) used an ELISA assay to measure affinities and found an apparent K_d_ ∼40 nM for control conditions that was reduced to an apparent K_d_ 5 nM in the presence of Mg^2+^ and Ca^2+^. Although the work provided valuable comparative values, as the authors comment, because the ELISA assay requires immobilization of one of the pairs, the K_d_ values do not necessarily reflect the K_d_ for binding in solution. The concentrations used in preparing the PhK/GPb sample (PhK tetramer 2.22 mg/ml [1.71 μM] and GPb dimer 1.08 mg/ml [5.54 μM]) correspond to 1 PhK (αβγδ):0.8 GPb dimer. Depending on the value of K_d_, saturation of PhK with GPb could have been between 1% and 80%. The appearance of the particles on the grids showed all particles with an increase in size, indicating that some were GPb bound, compared with the native PhK particles.

**Figure 4:**
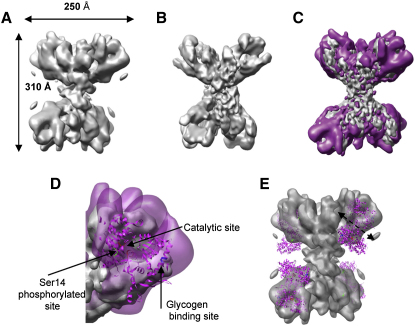
The 3D Structure of PhK Decorated with GPb (A) Surface representation of PhK/GPb at 18 Å resolution calculated from 18,704 frozen particles, butterfly orientation. (B) Surface representation of PhK 3D cryo-map filtered at 18 Å resolution. (C) PhK 3D cryomap at 9.9 Å resolution (in gray) shown with the difference map between PhK/GPb and PhK (in magenta). (D) Close-up view of the PhK 3D cryomap at 9.9 Å resolution (solid gray) shown with the difference map between PhK/GPb and PhK (transparent magenta) and the substrate GPb monomer (PDB 2GPB) in magenta. The N-terminal Ser14 phosphorylation site is indicated in green, the glycogen-binding site in blue, and the catalytic site in red. (E) Possible position of GPb dimers (magenta) bound to PhK. One subunit appears firmly bound and partially accounts for the density, whereas the other subunit projects out. The PhK/GPb surface is shown (transparent gray).

The 3D structure was calculated with 18,704 defocus-corrected particles and refined to 18 Å resolution as estimated from the 0.5 FSC cut-off. The final reconstruction is shown in Figure 4A. The threshold used to display the PhK/GPb model was chosen so as to give a compact particle with continuity that reflects the integrity of the particle as seen on the micrographs. The total number of particles used for the reconstruction of both models is similar (18,127 for the PhK and 18,704 for PhK/GPb), but for apo PhK, homogeneous particles were selected out of a total of 30,650 particles using the reference-free classification. Selection for PhK/GPb proved more difficult because of the lower number of particles and because the particles appeared to be naturally less homogeneous possibly because of incomplete binding of GPb. The nonhomogeneity is reflected in the lower resolution of the reconstructed image. The dimension of the overall structure is 310 × 250 × 200 Å and is greater than that of PhK alone (270 × 225 × 160 Å). The dimensions of the lobes of the butterfly-like shape are 250 × 120 Å showing a 25 Å increase in length from PhK, with generally more density at the ends of the lobes. The bridges have about the same length as for the PhK alone.

A difference map was calculated to localize more precisely the additional density arising from GPb bound to PhK. The PhK map was first filtered to 18 Å, the same resolution as for the 3D model PhK/GPb. Then the PhK and PhK/GPb maps were normalized and their difference computed. The extra density (shown in magenta in Figure 4C), presumably representing GPb, is located toward the end of each of the lobes of PhK. A possible manual fit of the atomic structure of the molecular mass of the 97 kDa GPb monomer (PDB 2GPB; Martin, 1990) into the additional density was performed. We positioned the GPb monomer so as to place Ser14, the site of phosphorylation, close to the position of γ subunit catalytic cleft. The location of GPb was subsequently adjusted using the docking program from CHIMERA (Goddard, 2007) (Figure 4D). In the crystal structure of GPb, the N-terminal region (residues 11–19) is mobile. Mobility around the site of phosphorylation in the native nonphosphorylated structure appears to be an important feature that allows the substrate to be accommodated in the defined extended conformation demanded by the kinase catalytic site (Lowe et al., 1997). The proposed docking mode for GPb to the kinase domain of PhK assumes some flexibility in the N-terminal region.

The lower volume of density seen for GPb in the PhK/GPb complex compared with the volume of density for the GPb dimer derived from the X-ray model indicates that only one subunit of GPb is localized, as observed previously (Venien-Bryan et al., 2002). The threshold chosen for the display of the difference map between PhK/GPb and PhK corresponds to a molecular mass of 620 kDa per PhK tetramer or 155 kDa per PhK monomer. The molecular mass of GPb monomer is 97 kDa or 194 kDa per dimer. Thus the density is sufficient to account for more than one GPb monomer but not sufficient for a dimer. The use of substoichiometric ratios of PhK:GPb (1 PhK [αβγδ] for 0.8 GPb dimers) could account for some low occupancy, but not the lack of the second subunit. If one subunit is firmly bound, then the other subunit must project into solution and is less well located and shows some flexibility. Figure 4E shows a possible position of the GPb dimer in the 3D model PhK/GPb. The fit is very approximate and complicated by the heterogeneity of the sample. In this view, the two-fold axis of the GPb dimer is approximately in the plane of the paper. Thus, the two-fold symmetry of GPb does not match the two-fold symmetry of PhK. Distance constraints indicate that it would not be possible for GPb to exploit the symmetry of PhK by having one GPb subunit bound to one lobe and the other subunit bound to the adjacent PhK lobe. The distance between the PhK catalytic sites on the lobes is 220 Å, whereas the separation of Ser14 sites on the two subunits of GPb is 62 Å.

### 3D Structure of the Smaller PhK Particles by Cryo-EM at 9.8 Å Resolution

**Figure 5:**
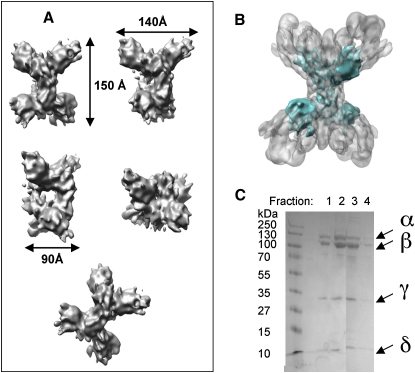
The 3D Structure of PhK Small Particles (A) Surface representation of the PhK at 9.8 Å resolution calculated from 28,284 selected frozen particles in the same orientations as in Figures 2A–2E. (B) 3D model of PhK reduced size (cyan), superimposed and centered on the 3D model of PhK normal size (in gray). (C) Four fractions of PhK after the Superdex column analyzed by SDS-PAGE followed by Coomassie blue staining.

Careful examination of particles selected from the micrographs indicated a mixture of large and smaller particles. The high-resolution 3D reconstructions described above for the larger particles could only be performed reliably if the two populations of particles were separated. For the smaller particles, the 3D reconstruction calculated from 28,284 particles led to the model at 9.8 Å resolution (0.5 FSC cut-off, Figure S3). The high resolution obtained reflects the homogeneity of particles selected. A gallery of some class averages of PhK small particles and the corresponding reprojections of the 3D map is shown in Figure S2. The EM model of the small particles is illustrated in Figure 5A with the same views as in Figures 2A–2E. The threshold used to display the PhK reduced size model was chosen so as to give a compact particle with continuity that reflects the integrity of the particles as seen on the micrographs. This volume corresponds to a molecular mass of about 500 to 550 kDa. The characteristic “butterfly-like” conformation is easily recognizable, but the size is clearly smaller and more compact (Figures 5A and 5B). The dimensions of the lobes in the projection are 140 × 40 Å compared to 225 × 110 Å for the larger particles. The dimensions of the overall structure are 150 × 140 × 90 Å compared with 270 × 225 × 160 Å for the larger PhK.

It has been known for some time that the α subunit is prone to proteolysis and loss leading to activation of PhK (Graves et al., 1973; Huston and Krebs, 1968; Trempe and Carlson, 1987). The most recent quantitative study by Hilder et al. (2005), which examined PhK proteolysis by caspase-3 in the context of ATP regulation during apoptosis, demonstrated the selective cleavage of the α subunit at residue Asp646 resulting in a 72 kDa and 66 kDa fragments and a 2-fold increase in activity. Therefore, we anticipated that the smaller particles might have resulted from proteolysis and dissociation of the α subunits.

After this work was completed, we analyzed the PhK preparation by analytical size exclusion chromatography (Figure S4) followed by SDS-PAGE gel electrophoresis (Figure 5C). The large and the small particles were not resolved into two peaks during Superdex 200 gel filtration, indicating that the subunits and the possible proteolytic fragments remained associated with the complex on S200 gel filtration (Figure S4). In the SDS gel (Figure 5C), dissociation and denaturation of the subunits results in a weakening of the band that represents the α subunit relative to that for the β subunit (and in comparison to Figure 1A), suggesting loss of proteolysed fragments. The band intensity ratio α/β ranges from 0.8 at the front of the peak to 0.5 for the tailing edge. This is in contrast to the gel for freshly prepared material, where the two bands corresponding to the α and β subunits are equally intense (Figure 1A). The gel electrophoresis suggests that the α subunit might have been partially proteolysed. This would be consistent with the higher ratio of activity (pH 6.8/pH 8.2) for calcium-activated PhK than is usually found. The lack of extended lobes in our 3D model of the smaller PhK particles also supports the loss of the α subunits. We propose that during specimen preparation for the EM studies, the proteolysed fragments are separated upon dialysis and dilution so that in the electron microscope we observe a mixture of (αβγδ)_4_ and (βγδ)_4_ or possibly (βδ)_4_ PhK particles. The SDS gel in Figure 1A was recorded immediately after preparation of the PhK, where there appears to be no proteolysis. The gel in Figure 5C was recorded several months later after initial tests and EM studies had been completed.

Comparison of Figures 3C and 5B suggests that the small particle might have also lost the γ subunit, although it is difficult to discern a relative weakening of the γ subunit band compared with that for the intact β subunit in the SDS gel (Figure 5C). If the γ subunit has also dissociated, it would remain in solution and contribute to the catalytic activity. In Figure 5C, the γ subunit runs at a molecular mass that is less than the 45 kDa observed for the intact γ subunit in Figure 1A. It has previously been noted that the γ subunit is sensitive to proteolysis between the kinase domain and the calmodulin-binding domain (Cox and Johnson, 1992, and references therein), releasing a kinase domain that is no longer sensitive to calcium dependence and is constitutively active. It is also possible that loss of the α subunits results in reorganization of the subunits so that the γ subunit locates at a different site, but we consider this less likely in view of the recognition properties of the PhK assembly.

The apparent difference in molecular mass between the large and small particles estimated from the volumes of the 3D EM maps is ∼750 kDa. The loss of four α subunits would correspond to a loss of 4 × 138 kDa = 552 kDa. The loss of the four γ subunits would correspond to a further loss of 4 × 44.7 kDa = 179 kDa. The loss of both the α and γ subunits would correspond to 730 kDa, roughly in agreement with the difference observed from the EM maps. However, the threshold level used to display the EM maps is partly empirical and quantitative estimates of molecular mass are approximate.

## Discussion

**Figure 6:**
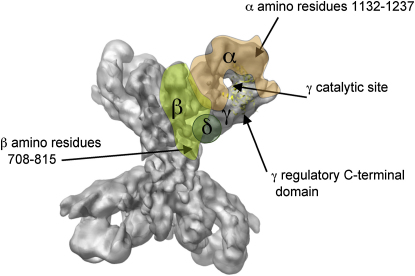
Schematic Location of the Four Subunits Inside the 3D Cryo-EM Volume of PhK

The PhK holoenzyme has an α_4_β_4_γ_4_δ_4_ stoichiometry and a corresponding molecular mass of 1.3 × 10^6^Da. Cross-linking and immunoelectron microscopy from the work of Carlson and colleagues have provided information for the possible subunit arrangement in the PhK holoenzyme. These results are summarized in Figure 6. The tetramers associate in a head-to-head arrangement with one (αβγδ)_2_ tetramer comprising each lobe of the “butterfly.” The lobes are connected by bridges that appear to be formed, at least in large part, by the β subunits. The important role for the β subunit in forming the quaternary structure of PhK is supported by various observations. (i) An epitope in the β subunit (residues 708–815) is localized to an interior position on the lobes near the interconnecting cross-bridges (Wilkinson et al., 1997). (ii) Selective proteolysis of the α subunit does not abolish bridges, whereas proteolysis of both β and α leads to the formation of unbridged single lobes (Trempe et al., 1986). (iii) An αγδ trimer has been produced by LiBr-mediated precipitation of the β subunit from the holoenzyme (Pickett-Gies and Walsh, 1985; Wilkinson et al., 1997). The αγδ complex is devoid of bridge elements as seen on electron microscope micrographs (Trempe et al., 1986). (iv) There are two bridges but four β subunits, suggesting the occurrence of β/β dimerization in an interlobal bridging; in support of this notion, cross-linking of the nonactivated holoenzyme with a short cross-linker results in the formation of a β/β dimer (Ayers et al., 1998). (v) Cross-linking experiments have shown that Lys303 from the γ subunit can form cross-links with Arg18 in the N-terminal region of the β subunit (Nadeau et al., 2007). The position of the γ subunit seen in the cryo-EM images is consistent with this.

The α subunit is the most susceptible of the four subunits to proteolysis by a number of proteases (Trempe and Carlson, 1987). It is presumed to be largely surface exposed and it is likely that the exterior lobes are largely composed of α subunits. This is supported by two findings. (i) The location of epitopes for monoclonal antibodies against the α subunit (amino residues 1132–1237) situated at the top of the lobes of PhK (Wilkinson et al., 1994) (Figure 6). (ii) Cross-linking experiments using transglutaminase have shown that small amounts of αα dimers are formed with activated PhK that are not observed with nonactivated PhK (Nadeau and Carlson, 1994). This finding suggests that the polypeptide backbones of the α-subunits stretch from the lobe tip to a more central location where they abut. Interactions between α and other subunits exist. The N-terminal ∼500 residues of the α-subunit contains a region that interacts with the β-subunit (Nadeau et al., 1998), and this later has a central location within each lobe (Figure 6). Cross-linking experiments have shown that the C-terminal region of the α-subunit (residues 1060–1237) interacts with the γ subunit (Nadeau et al., 1999) and an interaction between residues 724 and 981 of the α-subunit and the C-terminal domain of the γ-subunit has also been demonstrated (Harris et al., 1990).

In our new work, we have localized the γ subunit by polyclonal antibody binding and by molecular model fitting to the 9.9 Å resolution structure of PhK to a site on the interior of the lobes (Figure 3C and shown schematically in Figure 6). In the proposed location of PhKγ_t_, the γ subunit interacts with the other subunits in a way that is consistent with the cross-linking experiments. The C-terminal region of the γ subunit (residues 298–396) acts as an autoinhibitor of catalytic activity. In the presence of calcium, it is proposed that the interaction of the C-terminal region with the calmodulin-δ-subunit Ca^2+^ sensor serves to relieve inhibition. The interaction most likely involves the PhK calmodulin-binding region residues 343–366 (termed PhK5) (Cook et al., 2005; Dasgupta and Blumenthal, 1995; Dasgupta et al., 1989) and possibly the less conserved residues 302–326 (PhK13). There is currently no direct structural information for this C-terminal component of the γ subunit. The PhKγ_t_ located in our 3D model suggests that its C-terminal domain is oriented toward the bridge (Figures 3C and 3D). Nanogold labeling studies have shown that the δ subunit is located very close to the bridge (Traxler et al., 2001; dark green in Figure 6).

In our proposed model, only one GPb monomer from the dimer is firmly bound to PhK in the PhK/GPb complex. The other monomer apparently projects into the solution and is not visualized upon averaging. This arrangement would lead to the formation of GPab hybrids in which one subunit is phosphorylated and the other is not, at least transiently. There is supportive evidence (reviewed in Fischer et al., 1971), which suggest that partially phosphorylated GPab intermediates are produced that have properties intermediate between the non-phospho-GPb and the phospho-GPa states (Harris and Graves, 1990; Heilmeyer et al., 1970).

In our proposed PhK/GPb complex, GPb recognition by PhK could involve not only the local epitope around Ser14 but also other regions of GPb by other sites on the γ subunit and possibly other subunits such as the α subunits. Andreeva et al. (2002) have identified an interaction between the N-terminal region of GPb (residues 17–484), which lacks the phosphorylation site, and the regulatory α subunit of PhK (residues 864–1014). Both the holoenzyme PhK and the γ kinase subunit of PhK exhibit similar K_m_ for substrate GPb of 8–9 μM (Table 1). However, the kinase subunit has an almost 1000-fold higher K_m_ (1.8 mM) for a peptide substrate from GPb (Lowe et al., 1997), suggesting that even in the kinase subunit there might be remote docking sites that increase affinity of PhK for GPb. Similar results were observed in early work with the PhK holoenzyme (Tabatabai and Graves, 1978).

Both PhK and GPb are found localized on glycogen particles in muscle. The GPb glycogen-binding site is 30 Å away from the catalytic site (shown in Figure 4D). The entrance to the catalytic site is by a crevice that is able to accommodate four or five glucose units. It has been proposed that GPb is localized on glycogen through its glycogen-binding site and that the enzyme might be able to carry out the phosphorolysis of several glucose units in a processive mechanism before the enzyme must rebind the glycogen substrate. In our model, when complexed with PhK, the glycogen site of GPb is exposed and the site is located toward the top of the PhK lobes. Previous work has shown that PhK α subunits participate in the formation of the complex between glycogen and GPb via a direct interaction between the top of the lobes (α subunit) and the glycogen (Andreeva et al., 1999).

The observation that the PhK preparation contained a significant number of small particles in addition to the larger more conventional particles was surprising. A key step in the calculation of the high-resolution PhK structure was to select for a homogeneous class of particles. From the shape of the small particles, the calculated mass loss and the proposed location of the α subunit at the end of the lobes, we consider it possible that the small PhK particles have lost their α subunits. Partial selective loss of the α subunit from PhK particles observed on the SDS gel explains the higher than usual pH 6.8/8.2 activity ratio (0.125) observed in our activity measurements. The α and β regulatory subunits act as inhibitors of γ catalytic activity. Alleviation of inhibition occurs through phosphorylation, or by raising pH from 6.8 to 8.2 (which might mimic the increase in negative charge resulting from phosphorylation), or by proteolysis (Brushia and Walsh, 1999). The α subunit is targeted by most proteases and far more rapidly than the β subunit (Trempe and Carlson, 1987). Studies have shown that PhK is a substrate for caspase on induction of apoptosis and suggest that PhK activation by this route might be important for the generation of ATP during programmed cell death (Hilder et al., 2005). In addition, the 3D model for the smaller particles also indicates a possible loss of the γ subunit. Loss of the α subunits through proteolysis could weaken the association of the γ subunit with the particle, resulting in release of the catalytic γ subunit. The γ subunit also appears to have reduced molecular mass corresponding to the cleavage between the kinase and calmodulin-binding domains, which would result in a constitutively active kinase unregulated by calcium or the α and β subunits. Further work is required to establish this.

## Experimental Procedures

### Purification of PhK, PhK_γt_, and GPb

Nonactivated PhK from fast-twitch skeletal muscle of female New Zealand White rabbits was purified by the modified method of Cohen (1983) and Venien-Bryan et al. (2002). For details, see Supplemental Data.

### Measurements of Kinetic Parameters

The enzymatic activities of PhK and PhKγ_t_ were measured by monitoring the conversion of GPb to GPa (Cohen, 1983; Uyttenhove et al., 1991). In the absence of post-translational modifications, PhK exhibits optimal activity at pH 8.2 in the presence of Ca^2+^ and is almost inactive at pH 6.8 (Brushia and Walsh, 1999). The ratio of activities at pH 6.8 to pH 8.2 is a measure of the intact state of PhK, since modifications such as limited proteolysis and phosphorylation lead to greater activities at pH 6.8. PhK activity was measured at pH 6.8 and pH 8.2. For details, see the Supplemental data.

### Analytical Size Exclusion Chromatography of PhK

See Supplemental Data for details regarding analytical size exclusion chromatography of PhK.

### Antibody Studies

Polyclonal antibodies were raised in rabbits by Severn Biotech Ltd. UK. For details, see Supplemental Data.

### Specimen Preparation and Electron Microscopy

For cryo-EM, PhK was activated and glycerol or sucrose removed for optimal high-contrast conditions in vitrified ice by dialysis against 100 mM NaCl, 0.3 mM CaCl_2_, 5 mM MgCl_2_, and 50 mM HEPES (pH 8.2) for 2 hr. Five microliters PhK at about 1 mg/ml was placed on a 400-mesh copper grid coated with a thin holey-carbon film. The grid was blotted, vitrified by freeze plunging in liquid ethane, and transferred at liquid nitrogen temperature into the microscope using a side-entry nitrogen-cooled Gatan 626 cryoholder. All images were taken on a JEM-2100F (JEOL) using an acceleration voltage of 200 kV at a magnification of 50,000× with a defocus from −2.8 to −4.8 μm (nominal).

For the PhK/GPb complex, PhK (2.22 mg/ml) was incubated with GPb (1.08 mg/ml) overnight at 4°C at a molar ratio of 1 PhK (αβγδ) for 0.8 GPb dimers. Five microliters was deposited on a holey-carbon film grid, and the grid was washed by contact with a drop of dialysis buffer for 30 s, blotted, vitrified, and examined in the electron microscope as described above.

Micrographs of the negatively stained immunolabeled PhK were obtained with a Philips CM120 (FEI) at 100 kV. PhK, in a buffer of 200 mM NaCl, 0.5 mM CaCl_2_, and HEPES 50 mM (pH 8.2), was incubated with 4-fold molar excess of γ-antibodies for 90 min at room temperature to allow PhK-γ-subunit-antibody complex formation. The specimen was negatively stained using 2% uranyl acetate.

Images were recorded under low-dose conditions (10 electrons per A^2^) on Kodak SO 163 films.

### 3D Reconstruction

Particles were chosen via automated particle picking (Roseman, 2004) and data were processed with the SPIDER software package (Frank et al., 1996). The correction of the contrast transfer function was performed with the method of Wiener by filtering of volumes computed from focal series (Penczek et al., 1997). See Supplemental Data for details.

### Fitting of the γ Subunit and GPb onto the EM Model

Fitting of the atomic X-ray structure of the truncated kinase domain PhKγ_t_ (PDB 2PHK) into the cryo-EM map and the fitting of the atomic X-ray structure of GPb to the PhK/GPb map are described in Supplemental Data.

## Figures and Tables

**Table 1:** Kinetic Analysis of PhK

(A) Kinetic Parameters for PhK and PhKγ_t_ with GPb and ATP as Variable Substrates			
Enzyme (variable substrate)	k_cat_ (s^−1^)	K_m_ (μM)	k_cat_/K_m_ (s^−1^ μM^−1^)
PhK (GPb)	134 ± 3	9.3 ± 0.9	14.4
PhK (ATP)	97 ± 2.5	41.5 ± 4	2.3
PhKγ_t_ (GPb)	56 ± 0.7	8.5 ± 0.2	6.5
PhKγ_t_ (ATP)	44.5 ± 1.5	82.1 ± 12.4	0.54

GPb concentrations were varied between 0.4 and 7 mg/ml at saturating ATP of 0.9 mM; ATP concentrations were varied from 0.06 to 0.9 mM at GPb concentrations of 7 mg/ml.			

(B) V_max_ Values for PhK at pH 8.2 and 6.8			

Enzyme	Specific Activity		

Non-activated PhK pH 8.2	0.4 μmol/min/nmol		
Activated PhK pH 8.2	5.2 μmol/min/nmol		
Non-activated PhK pH 6.8	0.23 μmol/min/nmol		
Activated PhK pH 6.8	0.65 μmol/min/nmol		
Ratio non-activated PhK 6.8/8.2	0.575		
Ratio activated PhK 6.8/8.2	0.125		

Non-activated PhK: in the presence of 1 mM EGTA; activated PhK: in the presence of 0.5 mM Ca^2+^.

## Accession Numbers

The density map of PhK has been deposited in the Macromolecular Structure Database under the accession code EMD-1527.

## Supplemental Data

Document S1. Supplemental Experimental Procedures, Supplemental References, and Four Figures
