# Supplementary material for: The Structure of Phosphorylase Kinase Holoenzyme at 9.9 Å Resolution and Location of the Catalytic Subunit and the Substrate Glycogen Phosphorylase
Source: Structure. 2009 Jan 14;17(1):117–27. doi: 10.1016/j.str.2008.10.013 (PMC2639635; doi:10.1016/j.str.2008.10.013)
Supplement: Document S1. Supplemental Experimental Procedures, Supplemental References, and Four Figures [file mmc1.pdf]

Supplemental Data

The Structure of Phosphorylase Kinase Holoenzyme at 9.9 Å Resolution and Location of the Catalytic Subunit and the Substrate Glycogen Phosphorylase

Catherine Vénien-Bryan, Slavica Jonic, Vasiliki Skamnaki, Nick Brown, Nicolas Bischler, Nikos G. Oikonomakos, Nicolas Boisset, and Louise N. Johnson

SUPPLEMENTAL EXPERIMENTAL PROCEDURES

Purification of PhK, PhK$_{\gamma t}$ and GPb

Non activated PhK from fast-twitch skeletal muscle of female New Zealand White rabbits was purified by the modified method of Cohen (Cohen, 1983; Venien-Bryan et al., 2002). The procedures involved extraction at pH 9.0, homogenisation, centrifugation followed by precipitation at pH 6.1, ultracentrifugation, ammonium sulphate precipitation, gel filtration on a Sepharose 4B column, and final purification with a DEAE-cellulose column. In contrast to previous work we did not use a Superdex 200 column for the final purification as the material appeared suitable for EM work with no aggregation of particles after the DEAE cellulose step. PhK was stored frozen in 50 mM Hepes (pH6.8), 0.2 mM EDTA, 10% (w/v) sucrose. The kinase domain of the catalytic γ subunit (residues 1-298) PhKγ_t was expressed in E. coli cells as a GST fusion and subsequently purified (Cook, 2003). Glycogen phosphorylase b (GPb) was isolated from rabbit muscle according to the method of Fisher and Krebs(Fischer and Krebs, 1962) using 2-mercaptoethanol instead of L-cysteine and recrystallized at least four times. The enzyme was dialysed against buffer (25 mM β-glycerophosphate pH 6.8, 25 mM β-mercaptoethanol, 0.5 mM EDTA) and was freed of AMP by activated charcoal treatment prior to kinetic experiments. The concentrations of GPb and PhK were determined from absorbance measurements at 280 nm using an absorbance index $A^{1\%}_{1cm}$=13.2 (Kastenschmidt et al., 1968) and $A^{1\%}_{1cm}$=12.4 (Chebotareva et al., 2004) respectively.

Analytical Size Exclusion Chromatography of Phosphorylase Kinase

Approximately 20 μg of phosphorylase kinase was injected onto a Superdex 200 HR10/30 column at 0.5 ml/min in 50 mM Hepes pH 8.2, 200 mM NaCl, 0.5 mM $CaCl_2$, 5mM $MgCl_2$ using AKTA FPLC instrumentation (GE Healthcare). One peak was observed (Figure S4) near the exclusion volume, fractions of which were analysed by SDS-PAGE followed by coomassie blue staining. Densitometry of the α and β chain gel bands was performed with a Gel- Doc imager using Quantity-one software (BioRad Laboratories).

Measurements of Kinetic Parameters

AMP, ATP, glucose-1-phosphate (dipotassium salt), β-glycerophosphate, and glycogen were purchased from Sigma. Oyster glycogen was freed of AMP (Helmreich and Cori, 1964). The enzymatic activities of PhK and PhKγ_t were measured by monitoring the conversion of GPb to GPa by assaying phosphorylase activity in the presence of 10 μM AMP and 0.5 mM caffeine (Cohen, 1983; Uyttenhove et al., 1991) in the direction of glycogen synthesis. The volume of the reaction mixture was 0.2 ml and the reaction buffer was 50mM Hepes (pH 8.2), 0.5 mM $CaCl_2$, 2 mM DTT, 10 mM $Mg(CH_3CO_2)_2$ and 0.5 mg/ml bovine serum albumin. The concentration of GPb was varied from 0.4 to 7 mg/ml with saturating concentrations of ATP (0.9 mM). Similarly with respect to ATP the enzyme's activity was assayed at several concentration of ATP (0.06-0.9 mM) at saturating GPb concentration (7 mg/ml). EDTA was present in the stock GPb buffer (25 mM β-glycerophosphate pH 6.8, 25 mM β-mercaptoethanol, 0.5 mM EDTA) but after dilution for the kinase assays the maximum

concentration was 0.2 mM. After 1-3 min incubation of the reaction mixtures at 30° C the reactions were initiated by the addition of the PhK (78 ng/ml) or PhKγ_t (5-10 ng/ml). At time intervals 3.5, 7, 10.5 min the reactions were stopped by 50 times dilution to a buffer containing 100 mM triethanolamine/HCL (pH 6.8), 1 mM EDTA, 2 mM DTT at 0°C. In control experiments the kinase was replaced by buffer. GPa was assayed by measuring the release of orthophosphate from glucose-1-phosphate in a reaction mixture (0.3 ml) containing 50mM triethanolamine/HCL (pH 6.8), 0.5 mM EDTA, 1 mM DTT, 1%glycogen, 76 mM glucose-1- phosphate, 10 μM AMP and 0.5 mM caffeine. After 12 min the reactions were stopped in 0.33% sodium dodecyl sulphate. Free orthophosphate was measured as described(Fiske and Subbarow, 1925). Kinetic data were analysed with the non-linear regression program GraFit (Leatherbarrow, 1992).

In the absence of post-translational modifications, PhK exhibits optimal activity at pH 8.2 in the presence of Ca^{2+} and is almost inactive at pH 6.8 (Brushia and Walsh, 1999). The ratio of activities at pH 6.8 to pH 8.2 is a measure of the intact state of PhK, since modifications such as limited proteolysis and phosphorylation lead to greater activities at pH 6.8. PhK activity was measured at pH 6.8 and pH 8.2 in the absence of Ca^{2+} with 1 mM EGTA and in presence of 0.5 mM Ca^{2+}. The reactions were performed and progress curves were obtained up to 15 min. The concentration of GPb was 5.3 mg/ml and of ATP 0.9 mM. For reactions at pH 8.2 the PhK concentration was 78 ng/ml and at pH 6.8 the PhK concentration varied from 78 to 1200 ng/ml.

Antibody Studies

Polyclonal antibodies directed against the synthetic polypeptide (TAEEALAHPFFQQY), corresponding to the γ subunit residues 277-290, were raised in rabbits by Severn Biotech Ltd. UK. These residues are located in the C-terminal region on an exposed helix of the kinase domain. In an immunoblot analysis of the binding of the anti-γ antibody to denatured PhK, PhK (5 μg) was denatured in SDS-sample buffer and the subunits separated by SDS PAGE on a 12.5 % polyacrylamide gel. The protein was then transferred to a nitrocellulose membrane before being blocked with 100 mM Tris pH 7.4, 200 mM NaCl and 0.01 % Tween 20 (TBS) containing 3 % BSA for 1 hr. The PhK primary, anti-γ polyclonal antibody (10 mg/mL) was diluted 1/100 into TBS containing 3 % BSA and incubated with the membrane for 2 hr. The membrane was then washed 5 times in TBS at 10 minute intervals. The secondary goat, anti-rabbit-horseradish peroxidase linked secondary antibody (Pierce, USA) was diluted 1/5000 into BSA-supplemented TBS and then applied to the washed membrane for 1 hr with shaking. The antibody was then removed and the membrane washed a further 5 times in BSA supplemented TBS. The antibody-bound protein band was visualised with reagents from an enhanced chemiluminescence kit (Pierce, USA) and autoradiography.

In the PhK γ subunit kinase structure, residues 277-283 are located on the exposed C-terminal helix and are followed by residues 284-290 that are in an extended conformation. Only four of the 14 residues are partially buried (A281, V282, F286 and F287). Based on modeling of other calcium calmodulin dependent protein kinase structures, we envisage that this region will also be exposed in the intact γ subunit that contains the calmodulin binding regions. We do not know if the epitope is buried in the holoenzyme complex but the position of the truncated γ subunit located in the complex suggests that it could be external (Figure 3D). We did not perform immunoprecipitation studies to confirm that the antigenic site was available in the intact holoenzyme. However Wilkinson et al (Wilkinson et al., 1997) using a similar antibody raised to the same epitope and confirmed in ELISA assays that the antibody recognized intact PhK holoenzyme.

Three-Dimensional Reconstruction

PhK normal size: Ninety-three micrographs were digitized on a Super Coolscan 9000ED (Nikon, Japan) with a pixel size corresponding to 2.07 Å. Particles were picked automatically using FindEM program (Roseman, 2004) with the PhK 3D model calculated from negative stained images as a template (Venien-Bryan et al., 2002). The picked particles were

subsequently selected by hand and normalized making a total of 30,650 particles. Class averages were visualised and those classes producing averages inconsistent with the 3D model obtained from negative stained images were removed. The number of particles that remained after this step was 21,425. Some of the particle classes rejected corresponded to the 'small particles' (discussed later). The homogeneity of the population was verified using the principal component analysis in SPIDER(Frank, 1990). The defocus value for each micrograph and for the particle on it was measured using CTFTILT (Mindell and Grigorieff, 2003). The amount of astigmatism was checked for each particle, and the particle was discarded if the difference between its maximum and minimum defocus value was above 1,000Å. After this step, the number of particles was 19,174.
The three-dimensional reconstruction was computed with the 3D projection alignment procedures in SPIDER (Frank et al., 1996). For the starting model we used the 3D model calculated from negative stained images of PhK(Venien-Bryan et al., 2002) using the random conical tilt (RCT) series technique (Radermacher et al., 1987). The RCT approach is a robust 3D reconstruction algorithm that is based on pairs of images of the same specimen recorded under tilted and untilted conditions. The alignment of the cryo-EM images was refined with a library of reference projections in SPIDER. The reference projections were computed in each iteration using the previously computed 3D model, with 2° as the smallest angular step for calculation of the reference projection directions. The iterative refinements allowed the model to move from the initial structure guided by the cross correlation assessments with the observed particle images. The correction of the contrast transfer function (CTF) was performed with the method of Wiener filtering of volumes computed from focal series (Penczek et al., 1997). In this method, the data set is subdivided into defocus groups, for each group a 3D reconstruction is calculated, and the 3D reconstructions from the various groups are CTF corrected by Wiener filtering that combines them into a global 3D reconstruction. The Wierner filter correction peforms both amplitude and phase correction. Eight defocus groups were created using 2,000 Å as the value of the maximum difference for defocus values for particles to be assigned to the same group. After discarding the groups containing too few particles with respect to other groups, five groups remained, giving a total of 18,123 particles that were used for alignment and reconstruction. The smallest and largest numbers of particles per group were 2,809 and 4,948, respectively, and the smallest and largest average group defocus values were 21,422 Å and 28,961 Å, respectively. For each iteration, a mask with smooth edges and with the particle shape was applied on the 3D reference model. When the model had reached a resolution of ~12-15 Å, the masking of the reference model was preceded by sharpening to allow more precise fit to the surface volume. The sharpening was performed by applying a high-pass filter at 25 Å to remove coarser features and a subsequent low-pass filter at 8Å to sharpen higher resolution features. D2 symmetry was imposed during the reconstruction. The final volume was not subject to filtering. The resolution of the final 3D model is 9.9 Å as estimated by 0.5 Fourier-shell coefficient (FSC) cut-off.

PhK reduced size particles: The procedure for the 3D reconstruction of PhK reduced size particles was similar to that described as above. Particles were picked automatically using the same 3D template as above but the template was reduced in size by interpolation so it fits the reduced size PhK seen on the images. The picked particles were subsequently selected by hand and normalized. 29,631 homogeneous particles were kept after classification. Thirteen defocus groups were created using 1100Å as the value for the increment. 28,284 particles from eight selected defocus groups were used for the final reconstruction. The smallest and largest numbers of particles per group were 2,045 and 4.462, respectively, and the smallest and largest average group defocus values were 21,214 Å and 29,710 Å, respectively. No symmetry was applied during the reconstruction. The resolution for the final volume of the PhK reduced size reconstructed image is 9.8 Å as estimated by 0.5 FSC cut-off .

PhK-GPb: The procedure for the 3D reconstruction of PhK/GPb was the same as above. Particles were picked automatically from fifty eight micrographs using the PhK/GPb 3D model calculated from negative stained images as a template (Venien-Bryan et al., 2002). A total of 20,848 particles were subsequently selected by hand and normalized. Nine defocus

groups were created using 1000Å as the value for the increment. Seven groups remained, which made a total of 18,704 particles that were used for reconstruction. The smallest and largest numbers of particles per group were 1,049 and 3,668, respectively, and the smallest and largest average group defocus values were 18,805 Å and 26,244 Å, respectively. A mask but no sharpening was applied. A symmetry D2 was applied during the reconstruction. The resolution for the final volume of the PhK/GPb is 18Å as estimated by 0.5 FSC cut-off.

Fitting of the γ-Subunit and GPb onto the EM Model

Fitting of the atomic X-ray structure of the truncated kinase domain PhKγ_t (PDB 2PHK) into the cryo EM map, was performed with three different programs: i) Fourier space fitting using the operation 'fffear' from COOT (Emsley, 2004) ii) rigid-body fitting in Fourier space with Colores and Colacor algorithms from SITUS (Wriggers and Birmanns, 2001) and iii) real-space fitting with the FoldHunter algorithm from EMAN (Jiang, 2001). The three programs gave similar results for the fit of the subunit to the density. The fit was quantified with SITUS. The normalized correlation coefficients for the top four solutions corresponding to the fit of the PhK γ subunit to the four lobes were 1.0, 1.0, 0.94 and 0.92. The normalized correlation coefficients for the next four solutions were 0.89, 0.88, 0.83 and 0.83. The normalized correlation coefficients for all other solutions were < 0.75.

Fitting of the atomic X-ray structure of GPb was first done manually then adjusted the docking program from CHIMERA (Goddard, 2007) Molecular graphics images were produced using the UCSH CHIMERA package (Goddard, 2007) (Computer Graphics Laboratory, University of California, San Francisco, CA).

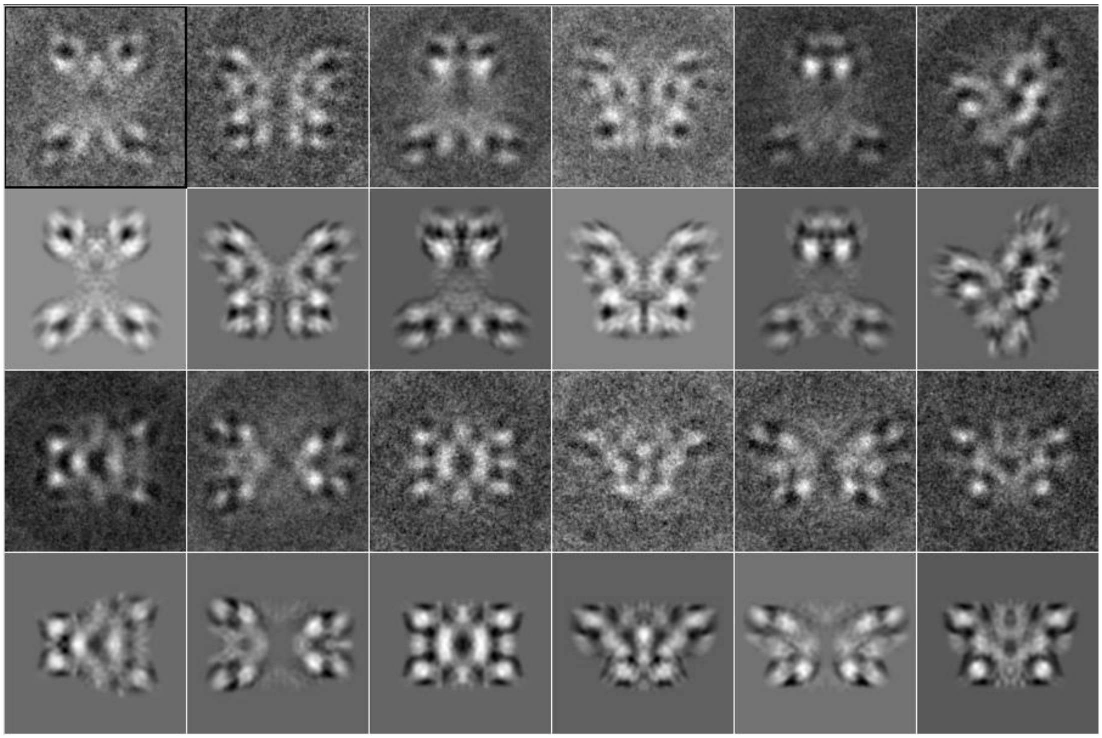

Figure S1. Class Averages of PhK Holoenzyme (rows 1 and 3) and the Corresponding Reprojections of the Three-Dimensional Map Below (rows 2 and 4)

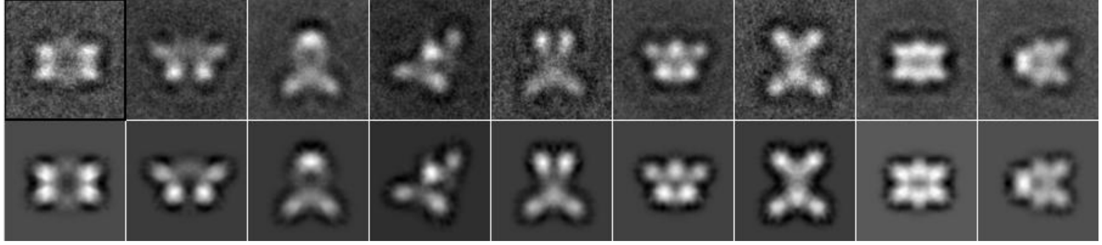

Figure S2. Class Averages of Phosphorylase Kinase Small Particles and the Corresponding Reprojections of the Three-Dimensional Map Below

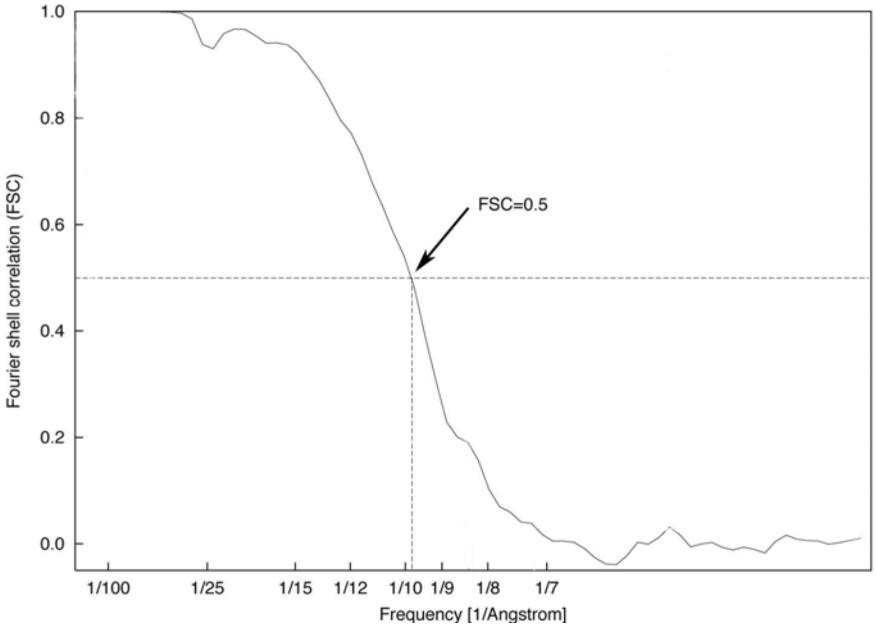

Figure S3. **Resolution of the PhK Small Particles**
Determination of the resolution of the final cryo 3D reconstruction of PhK FSC=fourier shell correlation. The resolution is 9.8 Å as assessed by 0.5 FSC cut-off.

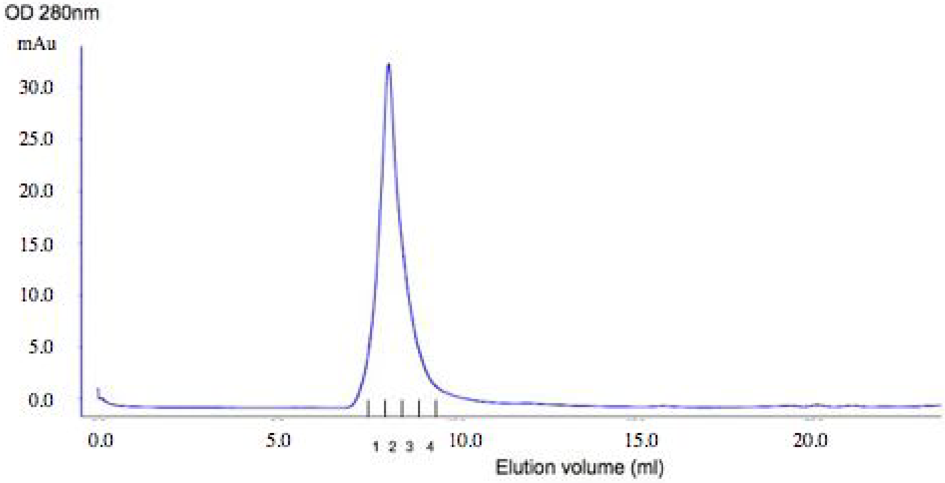

Figure S4. Analytical Gel filtration of Phosphorylase Kinase on Superdex 200 (10/30)

SUPPLEMENTAL REFERENCES

Brushia, R.J., and Walsh, D.A. (1999). phosphorylase kinase: the complexity of its regulation is reflected in the complexity of its structure. Front. Biosc. *4*, D618-641.

Chebotareva, N.A., Andreeva, I.E., Makeeva, V.F., Livanova, N.B., and Kurganov, B.I. (2004). Effect of molecular crowding on self-association of phosphorylase kinase and its interaction with phosphorylase b and glycogen. Journal of Molecular Recognition *17*, 426-432.

Cohen, P. (1983). Phosphorylase-Kinase from Rabbit Skeletal-Muscle. Methods in Enzymology *99*, 243-250.

Cook, A.G. (2003). Structural studies on the catalytic and regulatory mechanisms of protein kinases. DPhil thesis University of Oxford.

Emsley, P. (2004). Coot: model-building tools for molecular graphics. Acta Crystallographica Section D-Biological Crystallography *60*, 2126-2132.

Fischer, E.H., and Krebs, E.G. (1962). muscle phosphorylase b. In Methods enzymology, pp. 369-372.

Fiske, C.H., and Subbarow, Y. (1925). The colorimetric determination of phosphorus. Journal of Biological Chemistry *66*, 375-400.

Frank, J. (1990). Classification Of Macromolecular Assemblies Studied As Single Particles. Quarterly Reviews Of Biophysics *23*, 281-329.

Frank, J., Penczek, P., Agrawal, R.K., Grassucci, R.A., and Heagle, A.B. (2000). Three-dimensional cryoelectron microscopy of ribosomes. In Rna-Ligand Interactions Pt A, pp. 276-291.

Goddard, T.D. (2007). Visualizing density maps with UCSF Chimera. Journal of Structural Biology *157*, 281-287.

Helmreich, E., and Cori, C.F. (1964). The Role of Adenylic Acid in the Activation of Phosphorylase. Proc Natl Acad Sci U S A *51*, 131-138.

Jiang, W. (2001). Bridging the information gap: Computational tools for intermediate resolution structure interpretation. Journal of Molecular Biology *308*, 1033-1044.

Kastenschmidt, L., Kastenschmidt, J., and Helmreich, E. (1968). Subunit interactions and their relation to the allosteric properties of rabbit skeletal muscle phosphorylse b. Biochemistry *7*, 3590-3608.

Leatherbarrow, R. (1992). GraFit Version 3.0. Erithakus Software, Staines, UK.

Mindell, J.A., and Grigorieff, N. (2003). Accurate determination of local defocus and specimen tilt in electron microscopy. Journal Of Structural Biology *142*, 334-347.

Penczek, P.A., Zhu, J., Schröder, R., and Franck, J. (1997). Three-dimensional reconstruction with contrast transfer function compensation from defocus series. Scanning microscopy *11*, 147-154.

Radermacher, M., Wagenknecht, T., Verschoor, A., and Frank, J. (1987). 3-Dimensional Reconstruction From A Single-Exposure, Random Conical Tilt Series Applied To The 50s-Ribosomal Subunit Of Escherichia-Coli. Journal Of Microscopy-Oxford *146*, 113-136.

Roseman, A.M. (2004). FindEM - a fast, efficient program for automatic selection of particles from electron micrographs. Journal Of Structural Biology *145*, 91-99.

Uyttenhove, K., Bollen, M., and Stalmans, W. (1991). An Optimized Assay of Phosphorylase-Kinase in Crude Liver Preparations. Biochemical Journal *278*, 899-901.

Venien-Bryan, C., Lowe, E.M., Boisset, N., Traxler, K.W., Johnson, L.N., and Carlson, G.M. (2002). Three-dimensional structure of phosphorylase kinase at 22 angstrom resolution and its complex with glycogen phosphorylase b. Structure *10*, 33-41.

Wilkinson, D.A., Norcum, M.T., Fizgerald, T.J., Marion, T.N., Tillman, D.M., and Carlson, G.M. (1997). Proximal regions of the catalytic gamma and regulatory beta subunits on the interior lobe face of phosphorylase kinase are structurally coupled to each other and with enzyme activation. Journal of Molecular Biology *265*, 319-329.

Wriggers, W., and Birmanns, S. (2001). Using Situs for flexible and rigid-body fitting of multiresolution single-molecule data. J. Struct. Biol. *133*, 193-202.
